# Cetuximab plus 5‐fluorouracil in patients with advanced cutaneous squamous cell carcinoma: a retrospective cohort study

**DOI:** 10.1111/ddg.15695

**Published:** 2025-05-23

**Authors:** Julia Huynh, Thomas Eigentler, Rose K. C. Moritz, Gabriela Poch, Max Schlaak, Gabor Dobos

**Affiliations:** ^1^ Department of Dermatology Venereology and Allergology Charité – Universitätsmedizin Berlin corporate member of Freie Universität Berlin and Humboldt‐Universität zu Berlin Berlin Germany

**Keywords:** 5‐FU, anti‐EGFR, cetuximab, chemotherapy, cutaneous squamous cell carcinoma

## Abstract

**Background:**

For patients with locally advanced (la) or metastatic (m) cutaneous squamous cell carcinoma (cSCC) who are not candidates for curative surgery/radiation or systemic anti‐PD1 therapy, anti‐EGFR in combination with chemotherapy is a rational treatment option.

**Patients and Methods:**

We analyzed data from 20 patients with cSCC in this monocentric, retrospective study. 4/20 patients had laSCC and 16/20 patients had mSCC. Patients received combined cetuximab and 5‐FU between 2015 and 2023. Nine patients received cetuximab + 5‐FU as second‐line therapy (8 patients after anti‐PD‐1, 1 patient after radiochemotherapy).

**Results:**

One patient had partial response (PR) and 9/20 (45.0%) had stable disease (SD). Disease control rate (PR + SD) was 50%. No complete remissions were observed. One of the non‐responders suffered from laSCC, nine patients had mSCC with distant metastases (including parotid) and locoregional lymph node metastases. Treatment was well tolerated, with a median PFS of 3 months (95% confidence interval [CI] 2 months to not assessable [NA]) and median overall survival (OS) of 29 months (95% CI 11–NA). The most common adverse event was acne‐like rash in 40.0% of patients.

**Conclusions:**

For patients with advanced cSCC who are contraindicated to or have progressed on first‐line cemiplimab, combination of cetuximab and 5‐FU is a well‐tolerated but limited treatment option.

## INTRODUCTION

Cutaneous squamous cell carcinoma (cSCC) is the second most common skin cancer in fair‐skinned populations, with an increasing incidence. Chronic exposure to ultraviolet radiation, advanced age and prolonged immunosuppression are among the factors that increase the risk of developing SCC.^1^, ^2^, ^3^

Several clinical (location, horizontal tumor diameter and immunosuppression) and histopathological (depth of tumor infiltration, desmoplasia, degree of differentiation, perineural growth) prognostic factors for metastasis and disease‐specific survival are known.^4^

Treatment of cSCC follows the latest European and German guidelines.^5^, ^6^ For metastatic (m) and locally advanced (la) cSCC, immunotherapy with PD1 antibodies cemiplimab or pembrolizumab is recommended as first‐line systemic therapy for patients who are ineligible for curative surgery and/or radiotherapy.^7^ Immunocompromised patients following organ transplantation are at increased risk of metastasis. Systemic treatment with cemiplimab is contraindicated in these patients due to the potential for organ rejection or graft‐versus‐host disease.^8^ In these cases, or in patients who have failed anti‐PD‐1 therapy, alternative treatment options must be considered.

Several mono‐ and polychemotherapy protocols have been employed, but response rates and response duration remain unsatisfactory. Polychemotherapy appears to be more effective than monochemotherapy; however, it is associated with higher toxicity, which can be particularly limiting in this predominantly elderly patient population with multiple comorbidities. Platinum‐based therapy has been used as one of the standard chemotherapeutic options in the treatment of laSCC or mSCC.^10^

5‐FU‐based regimens appear to be a viable option for patients with advanced cSCC who are ineligible for or have progressed on PD‐1‐based therapy. Intravenous 5‐FU is a common treatment of colorectal cancer. Toxicity is dose‐dependent, making it also a candidate for older patients. Topical 5‐FU is widely used for actinic keratosis, a precursor of cSCC, with good response rates. There is also some evidence in the literature for 5‐FU in cSCC.^11^, ^12^, ^13^ However, there are no systemic chemotherapies approved for patients with laSCC or mSCC.^6^

The EGFR inhibitor cetuximab is recommended in combination with radiotherapy or platinum‐based chemotherapy for laSCC or mSCC of the head and neck. The combination of cetuximab and radiotherapy had a disease control rate of 92% and a response rate of 53%. However, the progression‐free survival (PFS) was only 5.7 months.^14^

Recent European guidelines recommend anti‐EGFR with chemotherapy or radiotherapy as off‐label second‐line treatment for patients with laSCC or mSCC.^6^ There are fewer data on combined anti‐EGFR and chemotherapy and, to our knowledge, no data on combined cetuximab and 5‐FU.

The aim of this retrospective study was to analyze data from 20 patients with laSCC or mSCC who received combined 5‐FU and cetuximab and to determine response rates, overall survival (OS) and PFS.

## PATIENTS AND METHODS

### Data collection

All cSCC patients (n = 23) who received 5‐FU and cetuximab between April 2015 and July 2023 were included in this study. Two patients had to be excluded due to missing data or loss to follow‐up. One patient with mucosal SCC was also excluded. In 3/20 patients, treatment was still ongoing at the time of data extraction. Patients were managed and treated according to European and German SCC guidelines, using the AJCC staging system for SCC, last updated in 2017.^5^, ^6^, ^15^

Clinical data on patient sex, age, date of diagnosis, tumor characteristics, prior and subsequent therapies, type and date of progression and death were collected by review of electronic patient records by clinically experienced dermato‐oncologists. If more than one SCC were diagnosed, the tumor with the highest risk profile for progression was used for tumor characteristics according to Brantsch et al.^4^ The stage at the start of 5‐FU/cetuximab therapy defined whether a patient was in the laSCC or the mSCC group. Adverse events were graded according to the *Common Terminology Criteria for Adverse Events* (CTCAE).^16^

The primary endpoint was overall survival, defined as the time from the first cycle of therapy to death (from cSCC or other causes) or to the date of last follow‐up at our hospital. Duration of response was measured in months from the start of 5‐FU/cetuximab therapy to the assessment of disease progression.

Our analysis was approved by the independent Ethics Committee of the Charité Universitätsmedizin Berlin (reference number: EA1/262/22).

### Therapy

The combined 5‐FU and cetuximab regimen was administered on an inpatient basis. Cetuximab was administered at a dose of 400 mg/m^2^ intravenously (i.v.) over 120 minutes on day 1 and 250 mg/m^2^ i.v. over 60 minutes weekly thereafter. 5‐FU 1000 mg/m^2^ over 24 hours was administered by Baxter elastomeric pump on days 1 and 2 over 24 hours every 3 weeks. Physiological sodium chloride solution was used to flush the infusion system. Patients were closely monitored for the risk of anaphylactic reaction. Dihydropyrimidine dehydrogenase (DPD) deficiency was ruled out before initiating treatment with 5‐FU.

We also performed daily inspections of the skin and oral cavity for mucosal changes, and body weight was assessed regularly. Patients were regularly treated with topical amphotericin B to prevent treatment‐related mucositis. The pre‐ and post‐therapy medications are shown in Table 1. During therapy, differential blood counts and liver and kidney function were monitored regularly.

**TABLE 1:** Pre‐ and comedication with every therapy cycle.

Dexamethasone 4 mg in 100 mL sodium chloride	Over 15 min. i.v.
Dimetindene 4 mg	Over 15 min. i.v.
Ondansetron 4 mg	Over 15 min. i.v.
Ondansetron 8 mg	Per os

### Statistical analysis

Statistical analysis was performed using SPSS version 27 (SPSS Inc., Chicago, IL, USA). The primary endpoints were OS and PFS. OS was defined as the time in months from the first cycle of cetuximab/5‐FU to death (from cSCC or other causes). Progression‐free survival was defined as the time in months from the first cycle of therapy to relapse or death. All events other than relapse were censored. If no event was recorded, survival was censored at the last follow‐up. Survival probabilities were estimated using the Kaplan‐Meier method. The reverse Kaplan‐Meier method was used to calculate the median follow‐up. The secondary endpoint was tolerability.

## RESULTS

### Patient cohort

Twenty patients were included in this study. All patients were male. The median age at first diagnosis was 74 years (range: 50–83). Half of the patients (50.0%) were immunosuppressed, five of whom had undergone kidney transplantation. Three patients were diagnosed with chronic lymphocytic leukemia, and one patient was diagnosed with osteomyelofibrosis. One patient had granulomatosis with polyangiitis and was treated with azathioprine.

In eleven patients (55%), the primary tumor was located in the chronically sun‐exposed head and neck area. In three patients, the stage at initial diagnosis was unknown. Most patients (45%) had AJCC stage III primary tumors.

At the start of treatment with 5‐FU and cetuximab, four patients (20%) had laSCC and 16 patients (80%) had mSCC. Locoregional lymph node metastases were present in 16 patients (80%). Distant metastases were seen in 14 patients (70%). Of these, two patients had distant lymph node metastases, one had distant skin metastases, six had lung metastases, six had parotid metastases, one had bone metastases, one had liver metastases, and one had splenic metastases.

Nine patients (45%) had received prior systemic therapy. One patient had undergone both chemotherapy and radiotherapy. Eight patients had received prior checkpoint inhibitor therapy, including seven who had received cemiplimab monotherapy and one who had received both cemiplimab and pembrolizumab. In all nine patients, prior therapies were discontinued due to progressive disease (PD).

Among the eight patients who received 5‐FU and cetuximab after progression with anti‐PD‐1 therapy, five had SD and one had a PR. Two patients experienced PD. Neither of these patients had a hemato‐oncological diagnosis. In one patient with PD, the squamous cell carcinoma was located in the head and neck area, while in the other, it was on the lower extremity.

In four patients of our cohort anti PD‐1 was contraindicated because of condition after organ transplantation. Three of them had SD, one had PD. All of them were in stable condition after kidney transplantation. Immunosuppression was carried out with mycophenolate mofetil, tacrolimus, and (methyl)prednisolone in the patient with PD as well as in two patients with SD. Time between organ transplantation and start of the therapy was < 10 years in the patient with PD and > 10 years in the patients with SD.

Patient characteristics are shown in Table 2.

**TABLE 2:** Patients’ characteristics.

Characteristic		n = 20 (%)
Localization primary tumor	Head/neck	11 (55%)
	Upper or lower extremity	3 (15%)
	Trunk	5 (25%)
	Occult	1 (5%)
AJCC Stage at first diagnosis^*^	Stage I	5 (25%)
	Stage II	2 (10%)
	Stage III	9 (45%)
	Stage IV	1 (5%)
Previous therapies	Surgery and/or radiotherapy	19 (95%)
	Chemotherapy	1 (5%)
	Checkpointinhibition	8 (40%)
Immunosuppressed		10 (50%)

* Unknown in three patients.

### Clinical Efficacy

All patients were treated for at least 1 week, 9/20 patients for 12 weeks or more. The median duration of treatment (DOT) was 9 weeks (min. 1 week, max. 77 weeks). One patient achieved a partial response. Stable disease was achieved in 9/20 (45%) patients. Stable disease was observed in three out of four patients with laSCC and six patients with mSCC. Four patients had distant (including parotid) and locoregional lymph node metastases and two patients had regional metastases only. The overall response rate was 5% and the disease control rate was 50%.

Ten patients (50%) had PD. One patient had laSCC, nine patients had mSCC with locoregional lymph node and distant metastases (including parotid). A summary of the data is shown in Table 3. There were a total of seven deaths (due to cSCC or death from other causes), none were due to adverse events.

**TABLE 3:** Treatment of cSCC with combined cetuximab and 5‐FU in 20 patients.

Pat. no.	La/mSCC	Immunosuppression	Prior therapies	Cycles cetuximab/5‐FU	Response	Time to progression in months	Follow‐up time in months
1	mSCC	–	Cemiplimab	43/16	SD	–	12
2	mSCC	KTX	Cemiplimab	47/17	SD	–	12
3	mSCC	KTX	None	10/3	SD	–	2
4	laSCC	Hem	Cemiplimab/pembrolizumab	36/2	SD	–	4
5	laSCC	KTX	None	14/6	SD	–	17
6	laSCC	AZA	None	7/3	PD	1	1
7	laSCC	KTX	None	2/1	SD	–	2
8	mSCC	–	None	12/4	PD	3	11
9	mSCC	–	None	9/3	PD	1	3
10	mSCC	CLL	None	8/3	SD	–	7
11	mSCC	–	Cisplatin + radiotherapy	6/2	PD	2	53
12	mSCC	–	None	5/2	PD	3	3
13	mSCC	–	None	7/3	PD	1	47
14	mSCC	–	None	11/2	PD	1	44
15	mSCC	KTX	None	9/3	PD	3	29
16	mSCC	–	Cemiplimab	16/6	PD	2	8
17	mSCC	–	Cemiplimab	7/3	PD	2	4
18	mSCC	CLL	Cemiplimab	10/4	SD	–	1
19	mSCC	–	Cemiplimab	15/5	PR	–	3
20	mSCC	CLL	Cemiplimab	15/5	SD	–	3

*Abbr*.: Pat, patient; no, number; KTX, kidney transplantation; CCL, chronic lymphocytic leukemia; AZA, Azathioprine

**FIGURE 1:**
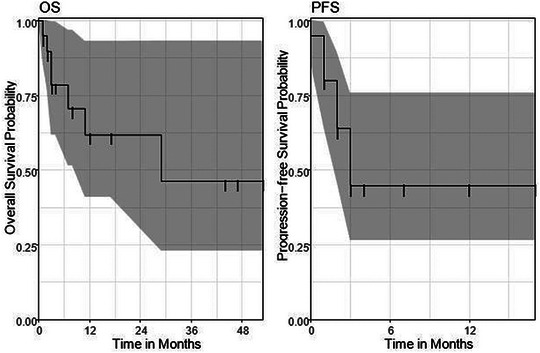
Overall survival (OS) and progression‐free (PFS) in months according to the total collective of patients with combined Cetuximab and 5‐FU in advanced cSCC.

Median OS was 29 months (95% CI 11–NA). The OS rate after 6 months was 79% (95% CI 62%–100%) and after 12 and 24 months it was 62% (95% CI 41%–93%). Median PFS was 3 months (95% CI 2–NA). The PFS rate after 6 and after 12 months was 45% (95% CI 26%–76%) (Figure 1).

The median follow‐up time was 12 months (95% CI 4–NA).

### Adverse Events

The most common adverse event was acneiform rash (8/20, 40.0% of patients). Five patients experienced grade 1–2 adverse events. Three patients had grade 3–4 skin toxicity and systemic treatment with doxycycline or isotretinoin was indicated in addition to topical treatment.

In one patient in the cohort, myelosuppression CTCAE grade 3 was documented, leading to treatment interruption because 5‐FU could not be excluded as the cause. There were no other adverse events reported.

## DISCUSSION

This is the first report of the use of combined 5‐FU and cetuximab in patients with advanced cSCC. We present our results on clinical efficacy and toxicity and compare them with other treatment options. The combination is an alternative for patients with contraindications to first‐line cemiplimab, such as immunocompromised patients or patients with progression after anti‐PD1, who represent a significant proportion of patients with advanced cSCC. Treatment options are very limited for these patient groups.

The median age at first diagnosis in our patient cohort was 74 (range: 50–83) years. This represents a predominantly older cohort of patients with SCC. Half of our patients were immuno‐compromised. Of these, 50% had undergone renal transplantation. Three patients were diagnosed with chronic lymphocytic leukemia and one patient had myelofibrosis, which was treated with ruxolitinib. Granulomatosis with polyangiitis was reported in one patient, who was treated with azathioprine. In addition to high cumulative UV exposure and fair skin type, immunosuppression is a risk factor for the development of non‐melanoma skin cancer.^17^, ^18^ Immunosuppression is also a prognostic factor for metastasis and disease‐specific survival. Other prognostic factors include vertical tumor infiltration depth, desmoplasia, degree of differentiation, perineural growth (histopathological factors), localization, horizontal and tumor diameter (clinical factors).^15^ Systemic treatment in immunocompromised patients can be challenging, as first‐line treatment with cemiplimab can lead to serious complications such as GvHD or organ rejection.^8^ For patients with contraindications to cemiplimab or progression on cemiplimab combination therapy, 5‐FU and cetuximab is an off‐label therapeutic option.^6^

We could not identify any clinical parameters which could predict response to cetuximab and 5‐FU in the eight patients who were progressive with anti PD‐1. Hemato‐oncological diagnoses or localization of the tumor in areas not chronically exposed to sunlight were not more frequent in the patients with PD than in the patients with disease control.

In our patient group receiving the combination therapy as first‐line treatment due to a history of organ transplantation, no clinical parameters predictive of therapy response could be identified. The immunosuppressive regimen in the patient with progressive disease did not differ from that of patients with stable disease. Notably, the interval between transplantation or initiation of immunosuppression and the start of therapy was even shorter in the patient with PD.

The PD1 antibody cemiplimab is recommended as first‐line therapy for patients with advanced SCC in the latest European guideline. Combined anti‐EGFR and radio/chemotherapy is recommended for patients with contraindications to immunotherapy.^6^ There are no randomized prospective trials comparing targeted therapies and chemotherapy in patients with cSCC. To the best of our knowledge, there are no retrospective or prospective data in the literature on the response rates of combined 5‐FU and cetuximab in cSCC. In our study, the response rate was 5%. One of our patients had PR and 9/20 had SD. The disease control rate was 50%. Median OS was 29 months (95% CI 11–NA) and median PFS was 3 months (95% CI 2–NA). Cetuximab + 5‐FU was the second line therapy after PD‐1 or radiochemotherapy in 45% of our patients, indicating that our patient population has fewer treatment options.

There is a wide range of data in the literature on mono‐ and polychemotherapy regimens. Response rates of chemotherapy for advanced SCC range from 17% to 84% in phase II studies in the literature.^10^, ^19^, ^20^, ^21^ Kramb et al. showed an overall response rate of only 17.4% in their patient population.^21^ Guthrie et al. showed in 28 patients treated with cisplatin‐based chemotherapy a complete response in 28% and a partial response in 40%. The median duration of response was 15 months.^10^

The antimetabolite 5‐FU, a pyrimidine analogue, is commonly used alone or in combination regimens in the treatment of gastrointestinal, breast and head and neck cancers. Capecitabine, its oral prodrug, is approved for the treatment of breast, gastric and colorectal cancer.^13^, ^15^, ^22^ Cartei et al. showed improvement in 9/14 patients with a median duration of 30+ months in their cohort of cSCC patients treated with oral 5‐FU.^11^ Response to chemotherapy is often limited with short OS and PFS, and toxicity may be a limitation as patients with SCC are often elderly and unable to receive aggressive polychemotherapy.

Montaudie et al. described a response rate of 53% and a disease control rate of 87% in SCC patients treated with cetuximab. In this study, cetuximab was used as first‐line therapy in most patients, which is not comparable to our patient cohort.^23^ Hillen et al. reported a response rate of 20% and Maubec et al. reported a response rate of 28% and a disease control rate of 69% in their phase II study of first‐line cetuximab.^24^, ^25^

In the study by Kamb et al. the overall response rate to cetuximab + chemotherapy was 14.3% in 14 patients. The median PFS of all patients (any therapy) in this retrospective study was 15 weeks, which is comparable to our results.^21^ Casassa et al. showed a median PFS of 6 months in their retrospective analysis of 14 patients with cSCC treated with cetuximab and paclitaxel, but toxicity was worse than in our cohort.^26^ Gold et al. showed an overall response rate of 10% and a disease control rate of 72% in a phase II study of 29 patients with cutaneous SCC treated with the EGFR inhibitor erlotinib. Prior therapies included radiation, surgery and chemotherapy.^27^

The Keynote‐629 study reported an overall response rate of 34.3% and a disease control rate of 52.4% in 105 patients treated with the PD‐1 antibody pembrolizumab, but 87% had received prior systemic treatment.^28^ Response rates for cemiplimab were significantly higher than for other systemic treatments for mSCC. Migden et al. reported the best overall response rate of 50% in the phase I cohort and 48% in the phase II cohort. Seven percent had a complete response (CR). Median disease‐free survival and overall survival were not reached at the time of data cut‐off.^29^ The use of immunotherapy in patients following organ transplantation and in patients with autoimmune diseases is still limited. In the review by Fisher et al. 37% of patients who received immune checkpoint inhibition experienced organ rejection and 14% died as a result of graft rejection.^30^ Less data are available for immunotherapy in patients with hematological malignancies such as chronic lymphocytic leukemia, but we have seen low response rates in these patients in our unit.^31^

The literature describes a worse outcome with cemiplimab in mSCC and laSCC in patients with a primary tumor in areas of the skin that are not chronically sun‐exposed, and cetuximab/5‐FU is an option for these patients.^32^

In our study cohort, adverse events were observed in 45.0% of the patients. Acne‐like rash was the most common adverse event in 8/20 patients. Dereure et al. described acne‐like rash in 57% of patients treated with cetuximab or cetuximab and cisplatin.^33^ Common cutaneous side effects of EGFR inhibitors are acne‐like eruptions, xerosis, and paronychia, which can significantly reduce patients' quality of life. Over 80% of patients treated with EGFR inhibitors develop papulopustular lesions on the face or upper trunk. Treatment options include topical acne or rosacea therapies, systemic antibiotics (doxycycline 200 mg daily or minocycline 100 mg daily) or isotretinoin (10–20 mg daily).^34^, ^35^ In their prospective study of 36 patients with advanced cSCC receiving cetuximab, Maubec et al. reported infections (22%) and bleeding (11%) as the most common adverse events.^25^

In our study, one patient had to discontinue treatment due to myelosuppression. Along with diarrhea and mucositis, myelosuppression is one of the most common side effects of 5‐FU chemotherapy.^36^ Cardiotoxicity of 5‐FU and capecitabine has also been described in the literature. Mild symptoms such as chest pain or hypotension occur in 0%–20% of patients, whereas severe symptomatic cardiotoxicity with cardiogenic shock occurs in ≤ 2% during fluorouracil treatment.^37^ No cardiovascular events were reported in our patients.

A limitation of this study is its retrospective design and the small number of patients included. In addition, tumor‐specific survival could not be accurately determined for all patients. Cause of death data were often missing, mostly because patients died outside our center and the exact cause of death was not known. In addition, the mostly elderly patients often have comorbidities, which further complicates the determination of the exact cause of death. Therefore, only OS was calculated.

## CONCLUSIONS

In our patient cohort, the combination of 5‐FU and cetuximab induced PR or SD in 50% of our patients. The median PFS was 3 months (95% CI 2–NA) and the median OS was 29 months (95% CI 11–NA). The most common adverse event was acne‐like rash in 40% of patients.

The combination of cetuximab and 5‐FU is a well‐tolerated treatment option for patients with advanced cSCC with contraindications or progression on first‐line systemic therapy with cemiplimab. Patients may benefit from this combination, which is also an option for older patients with comorbidities due to its low toxicity. Disease control rate was 50% in our cohort. Response rate was 5%. This fact shows that further randomized prospective data are needed on systemic treatment options for patients with advanced cSCC who are ineligible for anti‐PD1 therapy.

## CONFLICT OF INTEREST STATEMENT

None.
